# Proneural-Mesenchymal Transition: Phenotypic Plasticity to Acquire Multitherapy Resistance in Glioblastoma

**DOI:** 10.3390/ijms20112746

**Published:** 2019-06-04

**Authors:** Monica Fedele, Laura Cerchia, Silvia Pegoraro, Riccardo Sgarra, Guidalberto Manfioletti

**Affiliations:** 1 National Research Council (CNR), Institute of Experimental Endocrinology and Oncology “G. Salvatore” (IEOS), 80131 Naples, Italy; cerchia@unina.it; 2 Department of Life Sciences, University of Trieste, 34127 Trieste, Italy; spegoraro@units.it (S.P.); rsgarra@units.it (R.S.)

**Keywords:** epithelial–mesenchymal transition (EMT), glioma, tumor heterogeneity, phenotypic plasticity, chemoresistance, proneural-mesenchymal transition (PMT), glioblastoma

## Abstract

Glioblastoma (GBM) is an extremely aggressive tumor of the central nervous system, with a prognosis of 12–15 months and just 3–5% of survival over 5 years. This is mainly because most patients suffer recurrence after treatment that currently consists in maximal resection followed by radio- and chemotherapy with temozolomide. The recurrent tumor shows a more aggressive behavior due to a phenotypic shift toward the mesenchymal subtype. Proneural-mesenchymal transition (PMT) may represent for GBM the equivalent of epithelial–mesenchymal transition associated with other aggressive cancers. In this review we frame this process in the high degree of phenotypic inter- and intra-tumor heterogeneity of GBM, which exists in different subtypes, each one characterized by further phenotypic variability in its stem-cell compartment. Under the selective pressure of different treatment agents PMT is induced. The mechanisms involved, as well as the significance of such event in the acquisition of a multitherapy resistance phenotype, are taken in consideration for future perspectives in new anti-GBM therapeutic options.

## 1. Introduction

Glioblastoma (GBM) is the most common intrinsic and aggressive primary brain tumor in adults. It is characterized by alteration in crucial signaling pathways that results in the acquisition of hallmarks properties of cancer. Consequently, GBM exhibits a diffused infiltration throughout the brain rendering it incurable by surgery [1]. Since the 1970s, the first-line adjuvant treatment after surgery is represented by radiotherapy that has been recently been combined with targeted chemotherapy approaches involving DNA alkylating agents, such as temozolomide (TMZ). Unfortunately, this therapeutic option shows many limitations in its efficacy and almost all patients present the progression of the disease after a mean progression-free survival of 7–10 months [2,3]. Therefore, despite therapeutic strategies and supportive care having been improved, GBM remains characterized by a very poor quality of life and prognosis, with a mean median survival time of 15 months [2]. The poor outcome of GBM has been ascribed to the high degree of intra-tumor heterogeneity that occurs at multiple levels, including histopathological, transcriptional and genomic [4,5,6,7]. In the effort to facilitate the development of more effective target therapies, large-scale genomic projects on patient specimens have adopted to identify GBM molecular subtypes associated with prognostic values [8,9]. Ultimately, GBM has been classified into at least four molecular subgroups namely proneural (PN), neural (NL), classical (CL) and mesenchymal (MES) [9]. Since typical molecular alterations, sensitivity to therapy and prognosis are associated with each subgroup, this classification has implications in selecting target therapy strategies. The MES subtype is the most aggressive and strongly associated with a poor prognosis compared to PN subtype, in addition, a shift from PN to MES subtype can occur in patients following radiation therapy and chemotherapy [10]. The molecular events driving this transition are similar to those driving the epithelial-mesenchymal transition (EMT) in carcinomas cells. EMT has been described as a crucial mechanism by which carcinoma cells enhance their invasive capacity by losing cell polarity and intercellular adhesions acquiring a mesenchymal and more motile phenotype, thus promoting cancer metastasis [11]. The molecular mechanisms driving EMT have been investigated and highlight the down-regulation of E-cadherin together with other epithelial specification genes paralleled by the up-regulation of mesenchymal markers such as N-cadherin, vimentin and fibronectin [11,12]. Expression of mesenchymal markers by primary tumors correlates with enhanced invasiveness and poorer clinical prognosis. Moreover, mounting evidence associates EMT to chemotherapy resistance [13,14,15,16]. In general, cancer cell lines with epithelial features are more sensitive to chemotherapy drugs compared to those displaying a mesenchymal phenotype; on the contrary, the acquisition of resistance to several drugs is accompanied by the expression of mesenchymal markers [17,18]. Many signals that unleash EMT are involved in triggering and maintaining the mesenchymal state and converge in the activation of a network of master transcriptional regulators (EMT-TF) that drive the EMT process. Among these, a major role is played by ZEB 1/2, Snail 1/2/3, and Twist 1/2 [12,19]. The same EMT-TF network is also active in PMT and there is evidence, both in vitro and in vivo, supporting a critical role for these factors in the acquisition of the MES phenotype [20,21,22]. As for EMT, PMT has been shown to have a role in conferring an unfavorable prognosis. Specifically, radiation and chemotherapy induced resistance by promoting a PN to MES phenotypic shift, therefore investigation of PMT mechanisms is critical for improving therapy selection and patient outcomes.

## 2. Phenotypic Heterogeneity and Plasticity in Glioblastoma

**Figure 1:**
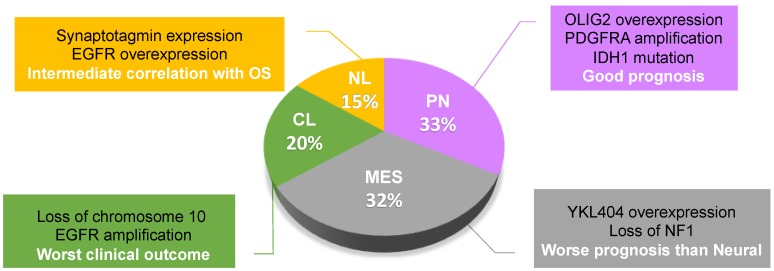
Glioblastoma subtype classification and prevalence of each subclass in all malignant gliomas. Some of the specific molecular and clinical characteristics are indicated on the side. OS, Overall Survival; NL, neural; PN, proneural; MES, mesenchymal; CL, classical.

GBM exhibits a high degree of phenotypic heterogeneity that molecularly corresponds to different gene expression signatures. Based on expression profiling studies, GBM has been subdivided into different molecular subtypes that have a prognostic value and delineate a pattern of disease progression [8,9,23]. One of the first pioneering studies in this sense is the work by Phillips et al., who defined three subclasses among high-grade gliomas, including PN, MES, and proliferative (Prolif) subtypes. The PN group expresses genes associated with the process of neurogenesis and is characterized by a better prognosis, while the MES and Prolif subtypes express genes of either cell proliferation or angiogenesis, respectively, and are both characterized by poor prognosis [8]. Subsequently, the deep molecular analysis by The Cancer Genome Atlas (TCGA) project has divided GBMs into four subclasses, including CL and NL, other than PN and MES [9]. Among all malignant gliomas, the most prevalent subtypes are PN (32.73%) and MES (32.4%), followed by CL (19.77%) and NL (15.09%) (Figure 1) [24]. Indeed, PN was largely observed in grade II and III gliomas, with a prevalence in oligodendrogliomas, while CL and MES were mostly observed in GBM [24,25]. According to recent comprehensive survival analyses, PN displays a good prognosis, NL has an intermediate correlation with overall survival, while MES and CL have the worst clinical outcomes [24,25].

Even though each subclass is associated with different mutations, such as platelet derived growth factor receptor A (*PDGFRA*) amplification in PN and loss of NF1 in MES, it is still not clear if these different subtypes are essentially different tumors or if they can evolve from each other. However, different subtypes can co-exist in the same tumor [6,7], and shifts in subtypes can occur. To our knowledge, there is no evidence of such shifts in low-grade gliomas that could justify a progression to high-grade gliomas (secondary GBMs). Indeed, a PMT associated to glioma progression from low- to high-grade gliomas is unlikely because secondary GBMs are characterized by a PN signature [26]. Conversely, the molecular signatures that define GBM subtypes are not a fixed feature of each tumor case, but may change after treatment and/or disease progression. Phillips et al. demonstrated that, upon recurrence, tumors tend to shift toward the mesenchymal phenotype reminiscent of EMT that is associated with increased malignant behavior of epithelial tumors. Particularly, they observed a loss of PN gene expression profile and a gain of the MES one, including frequent loss of OLIG2 expression and upregulation of YKL40 [8]. Indeed, MES phenotype is the hallmark of glioma aggressiveness and is strongly associated to poor prognosis. The ability of tumors to change subclass suggests that the tumor subtypes represent alternate differentiation states of the disease, but the unidirectionality of the shift PN-MES upon recurrence led us to hypothesize the acquisition of new genetic and epigenetic abnormalities toward the MES subtype. Accordingly, it has been recently shown that the PN gene *ASCL1* represses mesenchymal features by directly downregulating the expression of N-Myc downstream regulated gene 1, *NDRG1*. Abrogation of *ASCL1* or overexpression of *NDRG1* in PN glioma stem cells (GSCs) results in PMT, while overexpression of *ASCL1* in MES GSCs enhances their malignant features [27]. Different studies outlined that there is still a degree of heterogeneity within each molecular subclass. For example, within the PN group, we have previously shown that the expression of the transcriptional regulator PATZ1 is able to stratify patients in two subgroups with different overall and progression-free survival, in which higher levels of PATZ1 correlate with a longer survival [28]. Interestingly, the PN subgroup with lower levels of PATZ1 showed increased levels of the G-protein coupled receptor CXCR4, a well-known inducer of the mesenchymal phenotype in GBM [29,30], which has been shown to be downregulated by PATZ1 overexpression in GBM cells [28]. The trans-differentiation of GBM cells from PN to MES may imply a reprogramming process that leads differentiated cells to reacquire the capacity to differentiate into different cell types, i.e., to acquire stem-like features, for which OLIG2 has been shown to play a crucial role [31]. Indeed, there is a theory, developed by a computational methodology, that most GBM subtypes arise as, and evolve from, a PN progenitor [32]. The classification by TCGA highlights the association between treatment resistance and the stem cell phenotype, being the PN group (characterized by a stem-like signature) the only one fully unresponsive to aggressive chemo- and radio-treatment [9]. However, patients with a MES signature belong to the poorest prognosis subclass and are resistant to standard treatments [33], and PN tumors tend to shift toward the MES phenotype upon recurrence, or in response to radiation therapy, thus manifesting phenotypic plasticity [8]. Therefore, the tumor plasticity may render GBM cells more invasive or resistant to current therapies at different stages in their development [31].

Another theory, that has been applied as an explanation of the cancer plasticity in GBM, is the clonal variation, based on the clonal evolution model of cancer, which attributes the origin of clonal expansions to the accumulation of mutations and/or epigenetic changes. This is not contradictory with the stem cell theory, because genetic or epigenetic variations within the stem-like cells could be the culprits of the outgrowth of pre-existing resistant subpopulations upon treatment. Indeed, a single GBM can give rise to a variety of stem-like clones with different degrees of drug- and radio-resistance. The variation in multitherapy resistance is due to continual shifts along the PN-MES axis, associated with altered DNA methylation of MES transition regulators [10].

Finally, tumor microenvironment (TME) may also be a source of heterogeneity in cancer. A recent study proposed macrophages/microglia as an essential component that can regulate PN-MES transition in GSCs [34]. Consistently, the MES subtype of GBM exhibits a high degree of necrosis [35] and macrophage/microglia infiltration [36,37]. Bhat et al. demonstrated that the intratumoral PN-MES shift consequent to radiotherapy was linked to NF-κB activation and macrophages/microglia involvement in GBM [34]. More recently, the same group reported that this pro-inflammatory response transcriptionally regulates CD109 via C/EBPβ [38].

An integration of all these sources of heterogeneity could probably be the true explanation of tumor plasticity in GBM as in all types of cancer. GBM subtypes should not be considered stable phenotypes but dynamic states, which shift from one to another one upon treatment-dependent conditions in the microenvironment.

## 3. Signaling Mechanisms in PMT

**Figure 2:**
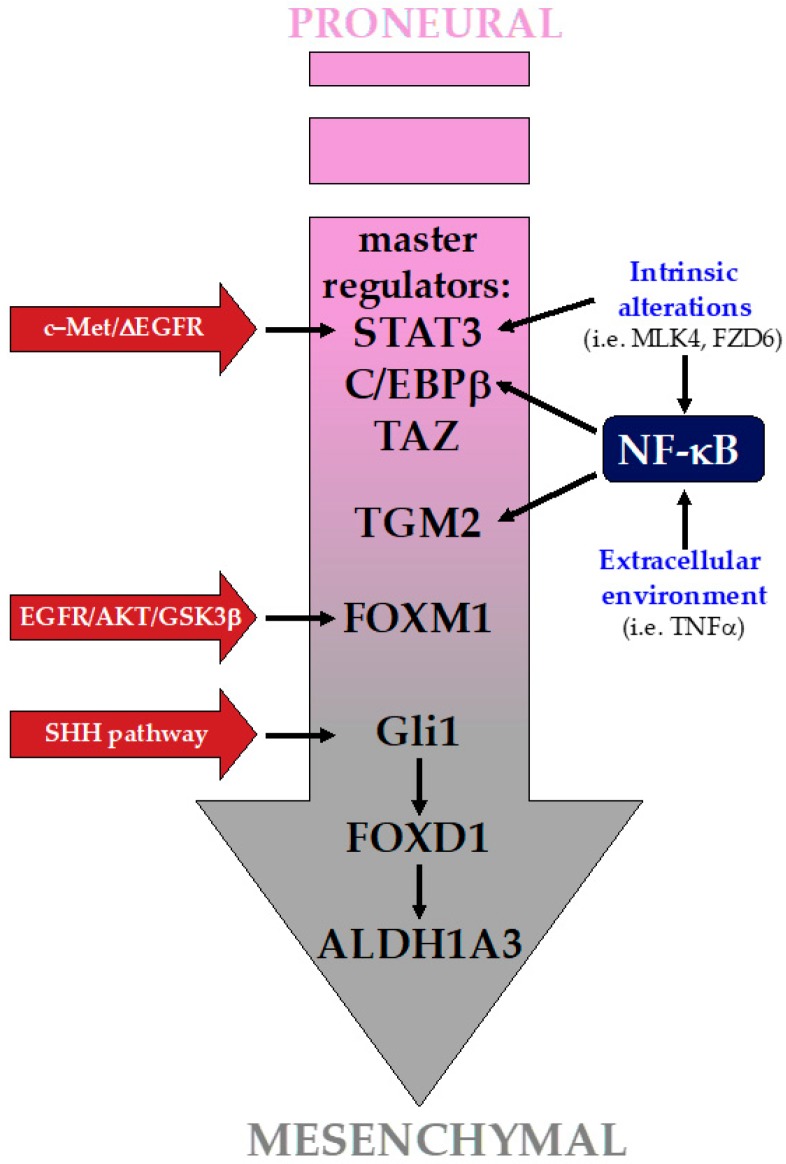
Signaling pathways in the proneural-mesenchymal transition (PMT). PMT is driven by the activation of a restrict set of master regulators (MRs), among which a key role is exploited by STAT3, C/EBPβ, and TAZ. NF-κB is an upstream regulator of these MRs, and several factors, both intrinsic and from the extracellular environment, can lead to its activation. Beside MRs, the activation of a plethora of factors by several distinct pathways play also a relevant role in driving the PMT.

The two GBM subtypes that appear consistently in the different molecular classification attempts are the PN and the MES groups, the latter being associated with a worst prognosis. In the following paragraph we will highlight some of the main signaling pathways and factors that are hijacked by GBM to acquire the MES phenotype (Figure 2).

### 3.1. STAT3 and C/EBPβ: Two Master Regulators

The research group of Iavarone [39], in a pioneer attempt to identify master regulators (MRs) of the mesenchymal gene expression signature (MGES), ends up with a list of six MRs (STAT3, bHLH–B2, C/EBP, FOSL2, ZNF238, and RUNX1), with STAT3 and C/EBPβ at the top of the hierarchical regulatory network. Specifically, they demonstrated that C/EBPβ and STAT3 were (i) responsible for MGES expression, (ii) able to reprogram neuronal stem cells towards a MES phenotype, and (iii) responsible for the aggressiveness traits of glioma cells. One of the most interesting hypotheses that emerged from their work is that the transforming ability of these two TFs could come from their opposing “normal” roles. The fact that STAT3 is an inducer of astrocyte differentiation while C/EBPβ push towards neurogenesis could constitute a conflicting situation that finally evolves driving neural stem/progenitor cells towards a MES phenotype.

STAT3 was demonstrated to be a key player in the acquisition of radiation-induced PMT. Indeed, it was demonstrated that after radiation regimens STAT3 turned out to be activated and notwithstanding its activation was temporary, it was sufficient to sustain a durable PMT [40]. The most interesting point was that by inhibiting JAK2, a STAT3 activator, by AZD1480 or ruxolitinib in combination with radiation, it was possible to prevent PMT and obtain a reduction in cell aggressiveness. Importantly, experiments performed with FVB/N mice allografted with PDGFRα^+^ proneural high-grade glioma cells showed that mice treated with AZD1480 in combination with radiation had a stronger benefit in terms of survival with respect to those subjected to radiation treatment alone, thus further sustaining a central role for STAT3 in PMT–dependent cell aggressiveness [40].

### 3.2. TAZ

In an attempt to further increase the number of MRs of the MES phenotype, the group of Aldape [41] found out other potential factors positively correlated with the MES signature, i.e., MAFB, HCLS1, and the two downstream factors of the Hippo pathway YAP and TAZ, that have been already demonstrated to play a role in the EMT in other cancer types. Authors showed that TAZ was not interdependent on STAT3 and C/EBPβ and vice versa, thus representing an alternative route contributing to the MES phenotype in GBM. They found that the TAZ promoter is hypermethylated in low-grade gliomas (grade II and III) with respect to grade IV ones (i.e., GBM), in which it is not methylated and, as a consequence, TAZ is expressed. They provided compelling evidences for a role of TAZ in driving the MES phenotype exploiting the RCAS/N–*tva* mouse model. Using this model, they overexpressed PDGF–B (a proneural tumor driver), TAZ, or PDGF–B plus TAZ in neural progenitor cells observing that mice overexpressing PDGF-B developed tumors with PN characteristics (predominantly grade II), those overexpressing TAZ were not able to drive tumor formation, but the combination of the two lead to the formation of grade III and IV tumors with a high expression of MES markers (FN1, CD44, and CTGF), thus providing an in vivo proof that TAZ is able to contribute to drive tumors towards a MES phenotype. Very recently, CD109 was identified as an upstream regulator of the YAP/TAZ pathway involved in the ionizing radiation (IR)-dependent PMT at the invading edge of GBM [38].

### 3.3. The Extracellular Environment and the NF-κB Pathway

A point that is still under debate is whether the PMT is an intrinsic process, or whether it is induced by factors that are present in the extracellular TME, or whether it is due to a combination of both. In support of a prominent role coming from the TME is the work by the laboratories of Sulman and Aldape [34]. In this study they showed that glioma sphere cultures from MES GBMs negative for the glioma CpG island methylator phenotype (MES/CIMP–GBMs) show an overall PN signature and that this was due to the in vitro lack of signaling from the TME. In an attempt to decipher the signaling pathways involved in this phenomenon they built up a TNF-α → NF–κB axis that controlled the MRs STAT3, C/EBPβ, and TAZ. They provided evidence that macrophages/microglia could be involved in inducing MES differentiation. This was further supported by immunohistochemical analyses of GBMs on serial paraffin–embedded sections in which it was shown that single GBMs displayed both PN and MES regions, and that the MES regions were positive for NF-κB activation and were in close contact with macrophages/microglia infiltration zones. However, the same authors discussed some cases where the phenotype of MES GSCs did not change upon removal from their TME, thus suggesting that also intrinsic mechanisms could be responsible for such an aggressive phenotype. It was later shown that the serine/threonine kinase MLK4 could be an important factor in the TNF-α → NF-κB axis [42]. MLK4 has been found highly expressed in MES GSCs and its expression was required to sustain the MES phenotype and to allow the growth of MES GSCs both in vitro and in vivo. Indeed, MLK4 binds and phosphorylates IKKα, which is involved in releasing NF-κB from its cytoplasmic inhibitor IκB, thus leading to NF-κB activation. In an immunohistochemical screening performed onto 87 high-grade gliomas specimens, the expression of MLK4 strongly correlated with that of CD44 (MES GBM marker) and turned out to be almost mutually exclusive with that of OLIG2 (PN GBM marker). More importantly, high MLK4 expressing patients displayed a shorter survival, thus indicating MLK4 as a predictor of worst prognosis in CD44-positive (MES GBMs) cases.

A very interesting work describing the relationship between extracellular stimuli and the PN/ MES phenotype, still converging onto the NF-κB and STAT3 pathways, is the one by the Cheng group [43]. In this study, the role of miR-125b and miR-20b in the modulation of the MES and PN phenotypes was highlighted. The authors demonstrated that the Wnt pathway induce the expression of these two miRNAs and that the Wnt pathway inhibitor FZD6 is one of their targets. FDZ6 is involved in the activation of an inhibitory pathway towards one of the final executors of the Wnt pathway, i.e., TCF4, thus blunting an autoregulatory activating loop between the Wnt pathway and miR-125b and miR-20b (Wnt → TCF4 → (miR-125b and miR-20b) —| FDZ6 —| TCF4). The most interesting point was that FDZ6, beside inhibiting TCF4 expression, was involved in the activation of a pathway (FZD6 → CAMKII → TAK1) leading to the activation of both STAT3 and NF-κB, factors that, as described above, play a key role in driving the MES phenotype. From one side, the Wnt pathway is responsible for the cell growth and self-renewal properties of PN GBM, on the other side, when blunted by FZD6, it allows the activation of a signaling boost towards a more aggressive phenotype.

Always related to the NF-κB pathway is the work by the Park group [44]. In this study they demonstrated that the perinecrotic regions of GBM display an upregulation of transglutaminase 2 (TGM2) expression, providing evidence that TGM2 is involved in the transition towards a MES phenotype; indeed, they demonstrated that, upon TGM2 silencing in MES GSCs, both MES MRs and MES markers were downregulated, while the contrary occurred in PN GSCs by TGM2 overexpression. These results were also confirmed in vivo using orthotopic mouse models. They showed that TGM2 is able to induce the degradation of GADD153, an inhibitor of C/EBPβ transcriptional activity. Furthermore, C/EBPβ expression was increased upon TGM2 overexpression and they provided evidences that the silencing of GADD153 was responsible for a strong induction of C/EBPβ. Authors demonstrated that radiation treatment induced TGM2 expression and TNF-α secretion was responsible for the activation of a signaling pathway leading to the activation of NF-κB through the downregulation of the NF-κB negative regulator IκBα. Importantly, they demonstrated that these findings were linked with a therapeutic opportunity: By generating an orthotopic xenograft mouse model implanted with MES GSCs, they demonstrated that treatment with the TGM2 inhibitor GK921 is able to improve survival and decrease the tumor size in mice. Furthermore, they showed that TGM2 inhibition overcomes the transition of cancer cells towards a MES phenotype that occurs upon radiotherapy, thus increasing the efficacy of the irradiation.

### 3.4. Multiple Routes to Drive the MES Phenotype

The Bogler group [45] found that STAT3 could be activated by the cooperation between two transmembrane receptors that are frequently overexpressed in GBM: The hepatocyte growth factor receptor (c–Met) and a constitutive active mutant of the epidermal growth factor receptor (∆EGFR/EGFRvIII). This signaling pathway relies on the hepatocyte growth factor (HGF) and forms a self–sustaining loop, i.e., HGF → c–Met/∆EGFR/EGFRvIII → STAT3/others → HGF, thus providing evidence to the presence of an autonomous autocrine loop.

FOXM1, a member of the Fox family of transcription factors, is involved in MES transition being responsible for the acquisition of mesenchymal features in glioma cells [46]. It was demonstrated that its action is due to the activation of the EGFR/AKT/GSK3β signaling pathway mediated by the FOXM1–dependent transcriptional activation of ADAM17. Interestingly, the EGFR/AKT/GSK3β pathway acts by stabilizing FOXM1 protein levels, thus constituting a self–sustaining loop [46]. It is relevant to underline that EGFR is well–known to be able to activate both the PI3K/AKT and Ras/MAPK pathways that exert a central role in driving tumor progression.

ALDH1A3 is highly expressed in MES GSCs, and ALDH1 inhibition by diethylaminobenzaldehyde attenuates the radiation-induced PMT of GSC [47]. Moreover, ALDH1A3 was shown to be a key player in driving the MES phenotype in GBM [48] and that the transcription factor FOXD1 regulates the transcription of the ALDH1A3 gene [49].

The hedgehog (HH) pathway has been widely involved in GBM [50], and in particular in controlling, in cooperation with the PI3K/Akt/mTOR pathway, several aggressive features of the GBM-initiating cells (GICs) [51]. In mouse cells, transcription of FOXD1 (also known as BF-2) was found to be induced by SHH (sonic hedgehog) stimulation and by Gli1 overexpression, a downstream effector of the SHH receptor [52], and Gli1 is known to be involved in GBM [50]. To our knowledge there are no data regarding the transcriptional regulation of FOXD1 by Gli1 in GBM, but it is conceivable that it could be under the control of SHH in this tumor as well. In addition, it was also demonstrated that factors able to stabilize the ALDH1A3 protein have a role in sustaining the MES phenotype. Indeed, this was the case of USP9X, a deubiquitinase, involved in the deubiquitination of ALDHA3, whose expression was responsible for self-renewal, tumorigenicity, and radio/chemoresistance of MES GSCs, and that was demonstrated to represent a promising pharmacological target for tumors expressing high levels of ALDH1A3 [53].

Although we are aware of not having provided a comprehensive review of all the mechanisms involved in the PMT, it appears evident that the MES phenotype relies on the activation of few MRs, but the pathways leading to their activation are multiples and other pathways can contribute to this process. A point which, at the moment, has not been clarified yet is whether GBM cells selectively hijack all these mechanisms or whether they somehow concur to drive the acquisition of a MES phenotype. Even though not yet replicated in humans, the various attempts to interfere singularly with each of these mechanisms led to promising results in terms of possibility to enhance the survival expectations of patients. However, the most interesting results could come by trying to adopt combinatorial therapeutic approaches.

## 4. PMT in Multitherapy Resistance

**Figure 3:**
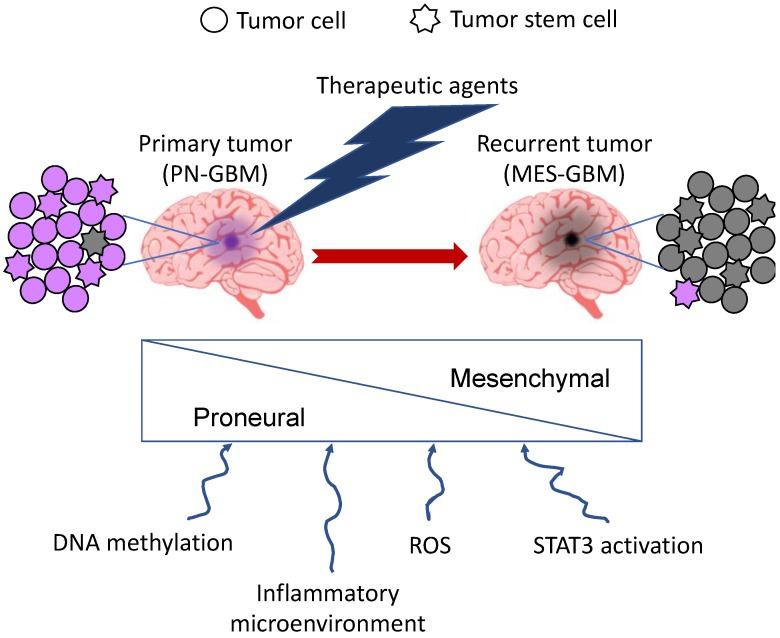
PMT upon radio- and chemotherapeutic treatment of GBM. A single GBM contains GSCs of both PN and MES phenotype. Upon treatment, most of them pass from the PN to the MES phenotype as a result of different treatment-dependent events, some of which are shown below. In purple: cells with proneural features; in grey: cells with mesenchymal features.

Current treatments for GBM, consisting of radiotherapy and concomitant adjuvant chemotherapy with TMZ, are still ineffective, since the tumor inevitably relapses and the prognosis is extremely poor, being fatal in 90% of patients 5 years after initial diagnosis [2,54]. A sub-population of tumor cells with stem-like properties, the GSCs or GICs, which are responsible for tumor initiation, is specifically endowed to resist or adapt to the standard therapies, leading to therapy resistance [54]. It has been recently proposed that GSCs can be sub-divided into two main groups, characterized by either a PN or a MES phenotype [55]. Segerman et al. have shown that a single GBM gives rise to many GIC clones with different resistance to therapies. However, they observed that, upon treatment with radiotherapy and different drugs, all clones become resistant to the same treatments acquiring a multitherapy resistance phenotype with different degrees of resistance along a continuous distribution, suggestive of a dynamic process in the GIC population [10]. The gene set enrichment analysis (GSEA) of these clones showed a clear connection of multitherapy resistance to MES signature on one side, and sensitivity to PN signature on the other side. Therefore, it is likely that a primary GBM contains both PN and MES GSCs, but the treatment pushes PMT in GSCs, resulting in a prevalence of MES GSCs in the recurrent tumor (Figure 3). The passage from PN to MES upon radio- and chemotherapy is governed by epigenetic changes, specifically DNA methylation, on regulatory elements of master regulators of the MES GBM subtype, such as FOSL2 [10,39]. It is likely that the inflammatory microenvironment consequent to the exposure to the therapeutic agents could be responsible for such dynamicity. More recently, Minata et al. reported that, upon exposure to IR, CD109 is highly induced, while CD133 is downregulated, in PN GSCs present at the invading edge of the tumor, which is often unresectable upon surgery. The resulting switch from PN to MES phenotype could explain the IR-dependent tumor recurrence. Mechanistically, IR activates NF-κB via ATM, which in turn induces CD109 via C/EBPβ. They also showed that CD109 acts upstream of the YAP/TAZ signaling pathway, which could contribute to the PMT of the CD109^+^ tumor cells [38]. However, due to the reversible nature of the process, elucidating the mechanisms of PMT may help to identify new strategies to sensitize GBM to treatment. Consistently, it has been shown that blockage of STAT3 activation is able to inhibit radiation-induced PMT, resulting in prolonged survival of mice with PN GBM [40]. Also, silencing of LncRNA-H19 was shown to decrease chemoresistance of human glioma cells to TMZ by suppressing PMT via the Wnt/β-catenin pathway [56].

Another therapy experimented for GBM is the antiangiogenic therapy (e.g., bevacizumab/anti-VEGF) that, by reducing vascular permeability is thought to reduce the tumor burn of a highly vascularized cancer such as GBM. However, after an initial delay in tumor progression, chronic exposure to antiangiogenic therapy ultimately provoked an aggressive treatment-resistant phenotype [57]. Also, in this case, gene expression and GSEA of resistant clones showed an increase in genes associated with the MES GBM subtype, thus identifying a PMT in tumors resistant to antiangiogenic therapy [58].

Upregulation of MES markers with concomitant downregulation of the PN phenotype, consequent to increased intracellular levels of reactive oxygen species (ROS), was also observed in primary GSCs treated with the cannabinoid and redox modulator cannabidiol (CBD), thus suggesting a combinatorial approach consisting of CBD and small molecule inhibitors of ROS for a more successful treatment of GBM [54].

## 5. Conclusions and Therapeutic Perspectives

**Figure 4:**
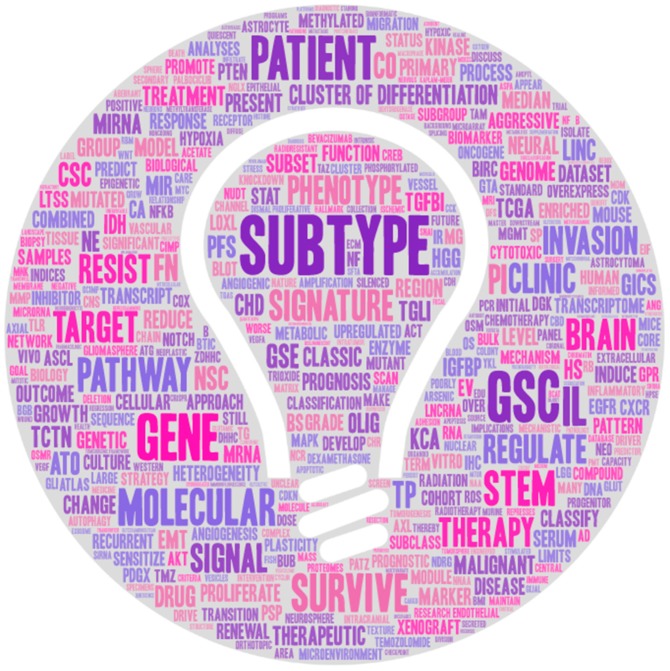
Word cloud image obtained using all published abstracts of the last 5 years searched with GBM, Proneural, and Mesenchymal as keywords. The resulting message highlights the importance of the subtype signature and molecular pathways, especially those involved in GSC regulation, resistance and invasion, for patient’s target therapy.

It seems clear that in order to combat GBM, there must be a shift from classical cytotoxic chemotherapy to more targeted therapies, taking into account the high degree of heterogeneity and molecular complexity of the tumor. Subtyping GBM is a key process in order to impact a patient’s survival providing them targeted therapies. Gene molecular signatures are features associated with specific tumor phenotype. The definition of pathways and key molecules involved in tumor signaling, especially those involved in GSC resistance and invasion, will provide therapeutic opportunities that could be translated into GBM treatment clinics (Figure 4).

Despite a continuous effort to design new targeted drugs, such as molecular pathway inhibitors, these molecules often result in toxicity and acquired resistance. The high level of cross-stimulation among the targets of the new therapeutic agents poses a major obstacle to their successful development. Blocking one target often allows others to act as salvage mechanisms for cancer cells. The case of the constitutively active EGFRvIII mutant is a paradigmatic example. EGFRvIII expression influences multiple aspects of GBM biology, including the rising of glioma stem-like cells and maintenance of tumor heterogeneity [59], thus encouraging the development of therapeutics to block its activity. Nevertheless, GBM may escape from EGFR-targeted therapies by the occurrence of alternative kinase signaling pathways, as those dependent from PDGFRβ and MET, which compensate the pharmacological perturbations [60,61]. Therefore, testing combinations of diverse targeted drugs and identifying key biomarkers to improve selection of patients most likely to benefit, is an essential issue in the novel treatment modalities against GBM.

Notably, in addition to GBM heterogeneity, including tumor cell plasticity as the source of cancer stem cells, the existence of the blood-brain barrier (BBB) represents an important limitation to effective therapy. GBMs are indeed “protected” by their localization beyond the BBB, which limits the penetration of many antineoplastic drugs [62]. A variety of approaches are currently under investigation for overcoming the drug delivery issues including invasive techniques, e.g., BBB transient disruption, intracerebroventricular and intrathecal infusion, and non-invasive techniques, e.g., inhibition of multidrug efflux transporters and various pharmacological strategies for modifying drugs to facilitate their transport across the BBB. Among the last ones, targeted drug delivery nanovectors are receiving increasing attention for their ability to transport drugs within the cerebral tissue with minimal off-target toxicity. Drug-loaded nanosystems conjugated to targeting agents as peptides, antibodies, or oligonucleotide aptamers are now at the forefront of innovation [63,64].

In conclusion, it is important to understand the impact of a treatment on the biologic response and selective pressure within the tumor and its subsequent behavior, in order to make changes in the treatment regimen that could target a tumor that has evolved into a different and more aggressive form. In particular, since it is clear that resistance is associated with a MES-like cell state of the GICs, specific inhibitors of the MES phenotype in combination with the standard therapeutics could synergistically inhibit GBM progression and should be considered for the development of novel therapeutics.

## Abbreviations

GBM	Glioblastoma
PMT	Proneural-Mesenchymal Transition
TMZ	Temozolomide
PN	Proneural
MES	Mesenchymal
NL	Neural
CL	Classical
Prolif	Proliferative
EMT	Epithelial-Mesenchymal Transition
TCGA	The Cancer Genome Atlas
MR	master regulator
MGES	mesenchymal gene expression signature
TME	tumor microenvironment
GSC	Glioma stem cell
IR	Ionizing Radiation
G-CIMP	glioma CpG island methylator phenotype
GIC	Glioma-initiating cell
GSEA	Gene Set Enrichment Analysis
ROS	reactive oxygen species
CBD	cannabidiol
BBB	Blood-brain barrier
